# 

**Published:** 1889-12

**Authors:** 


### CONTENTS:

Article I.—Georgia State Dental Society. Reported by Mrs. M. W. J. - - - - - - - 337

" II.—A study of the Chemical Composition of the Dental Pulp. By W. H. Whitslar, D. D. S., M. D. - 350

" III.—Case of persistent Facial Neuralgia of Reflex Dental Origin due to an Unerupted Cuspid Tooth. By Edward C. Kirk, D. D. S. - - - - 356

" IV.—Failures. By. Dr. C. R. Butler. - - - 359

" V.—An Address on Anæsthetics. By Dudley W. Buxton, M. D., C. S. - - - 362

" VI.—One way of Increasing a Dentists Usefulness. By William D. Kempton, M. D., D. D. S. - 368

" VII.—To Whom it may Concern. - - - - - - 372

The Dedication of Meharry Dental and Pharmaceutical College 378

### EDITORIAL.

The Mouth in Backward Children (Imbecile) of the Mongolian Type. - - - - - - - - 379

### MONTHLY SUMMARY.

Gonorrhœa at Five Years of Age. - - - - - - 383

Death from Chloroform. - - - - - - 384

